# A machine learning model based on emergency clinical data predicting 3-day in-hospital mortality for stroke and trauma patients

**DOI:** 10.3389/fneur.2025.1512297

**Published:** 2025-03-19

**Authors:** Xu Chen, Bin Yu, Yaming Zhang, Xin Wang, Danping Huang, Shaohui Gong, Wei Hu

**Affiliations:** ^1^Shangrao People's Hospital, Shangrao, China; ^2^Shangrao Municipal Hospital, Shangrao, China; ^3^Huaian Hospital of Huaian City, Huai'an, China; ^4^School of Nursing, Jinzhou Medical University, Jinzhou, China

**Keywords:** stroke and traumatic brain injury, in-hospital mortality prediction, machine learning algorithms, emergency scoring systems, predictive models

## Abstract

**Background:**

Accurately predicting the short-term in-hospital mortality risk for patients with stroke and TBI (Traumatic Brain Injury) is crucial for improving the quality of emergency medical care.

**Method:**

This study analyzed data from 2,125 emergency admission patients with stroke and traumatic brain injury at two Grade a hospitals in China from January 2021 to March 2024. LASSO regression was used for feature selection, and the predictive performance of logistic regression was compared with six machine learning algorithms. A 70:30 ratio was applied for cross-validation, and confidence intervals were calculated using the bootstrap method. Temporal validation was performed on the best-performing model. SHAP values were employed to assess variable importance.

**Results:**

The random forest algorithm excelled in predicting in-hospital 3-day mortality, achieving an AUC of 0.978 (95% CI: 0.966–0.986). Time series validation demonstrated the model’s strong generalization capability, with an AUC of 0.975 (95% CI: 0.963–0.986). Key predictive factors in the final model included metabolic syndrome, NEWS2 score, Glasgow Coma Scale (GCS), whether surgery was performed, bowel movement status, potassium level (K), aspartate transaminase (AST) level, and temporal factors. SHAP value analysis further confirmed the significant contributions of these variables to the predictive outcomes. The random forest model developed in this study demonstrates good accuracy in predicting short-term in-hospital mortality rates for stroke and traumatic brain injury patients. The model integrates emergency scores, clinical signs, and key biochemical indicators, providing a comprehensive perspective for risk assessment. This approach, which incorporates emergency data, holds promise for assisting decision-making in clinical practice, thereby improving patient outcomes.

## Introduction

Stroke and TBI are major causes of death and disability worldwide, placing a significant burden on public health and healthcare systems (1). The first 72 h after hospital admission represent a critical window for patients with stroke and traumatic brain injury, during which the risk of mortality is particularly high due to complications such as cerebral edema, hemorrhagic transformation, and secondary brain injury (2). Accurate prediction of mortality risk within this crucial period is vital for several reasons: It enables early identification of high-risk patients who may require more intensive monitoring and aggressive intervention; It facilitates timely medical decision-making, including the need for surgical intervention or admission to intensive care units; It allows for more efficient allocation of medical resources and specialized care; and It provides objective evidence for early communication with families regarding prognosis and treatment options (3). Studies have shown that early risk stratification and subsequent targeted interventions can significantly improve patient outcomes and potentially reduce mortality rates. Moreover, accurate prediction of short-term mortality risk helps healthcare providers implement preventive measures for specific complications and optimize treatment protocols, ultimately contributing to improved quality of emergency care. Accurately predicting the short-term mortality risk for these patients is crucial for optimizing treatment strategies, resource allocation, and improving patient outcomes (4). In recent years, the application of machine learning in the medical field has become increasingly widespread, particularly showing significant potential in the development of predictive models (5). Multiple studies indicate that machine learning algorithms outperform traditional statistical methods in predicting outcomes for stroke and trauma patients (6). Several machine learning models have been developed for predicting outcomes in stroke and TBI cases. For stroke, existing models primarily focus on predicting long-term functional outcomes (measured scores at 3–6 months), mortality beyond 30 days, and rehabilitation potential. These models typically utilize clinical variables, imaging features, and demographic data (7). Regarding TBI -related research, machine learning models have been designed to predict various outcomes, including Glasgow Outcome Scale (GOS) scores, the need for intensive care, and long-term survival rates. These models often incorporate neurological examination findings, CT characteristics, and biochemical markers. However, these existing models predominantly target long-term outcomes or specific complications, rather than acute phase mortality prediction. Furthermore, most models focus exclusively on either stroke or TBI, lacking the capability to address both conditions within a single predictive framework. However, most existing models primarily focus on long-term prognoses, with relatively few studies addressing the prediction of mortality risk during the acute phase (such as within the first 3 days of hospitalization) (8). Emergency clinical data, including vital signs, laboratory test results, and scoring systems like NEWS2 and GCS, have proven to be effective indicators for predicting patients’ short-term prognosis (9, 10). Machine learning models that incorporate these data have the potential to offer more accurate and timely risk assessments (10, 11). Recent research shows that ensemble learning methods, like Random Forests and XGBoost, excel in managing complex medical data (12, 13). Additionally, using interpretative techniques such as SHAP (shapley additive explanations) has clarified the decision-making processes of complex models, thus improving clinical interpretability (14).

Currently, there is a lack of comprehensive predictive models specifically for in-hospital mortality within 3 days for stroke and trauma patients (15). Developing such a model could fill gaps in existing research and provide valuable support tools for emergency medical decision-making (16).

## Methods

### Research population

This retrospective study included 2,125 patients who visited the emergency department of two top-tier hospitals in China from January 2021 to March 2024, among whom 937 were stroke patients and 1,188 were brain injury patients. Inclusion criteria comprised: age ≥ 18 years, emergency department visit, confirmed diagnosis of stroke or brain injury, and complete baseline admission data and biochemical examination results. Exclusion criteria included: age < 18 years, non-emergency visit, already in a terminal state upon admission, incomplete data, transfer to another hospital within 48 h, or refusal to allow data to be used for research.

### Baseline data

Gather baseline data from the electronic medical record system, including demographic details (age, gender), lifestyle habits (smoking, alcohol use), clinical characteristics (bowel habits, sleep patterns, metabolic syndrome, admission time, surgery status), laboratory test results (blood urea nitrogen, creatinine, albumin, potassium, total bilirubin, aspartate aminotransferase), and clinical scores (NEWS2 score, Glasgow Coma Scale—GCS). The primary outcome is in-hospital mortality within 3 days of patient admission (see Table 1 for baseline information).

**Table 1:** Neurological signs upon emergency admission for patients with cerebrovascular accident (stroke) and traumatic brain injury.

Characteristic	Survivors (*n* = 1709)	Non-survivors (*n* = 416)	*P*-value
Male (%)	1,076 (63.0%)	273 (65.6%)	0.339
Age, years, mean (SD)	61.4 (12.1)	60(21.9)	0.76
Stroke (%)	781(83.4%)	156(16.6%)	<0.001
TBI (%)	928(78.1%)	260(21.9%)	<0.01
Current smoker, *n* (%)	448 (26.2%)	120 (28.8%)	0.305
Alcohol consumption, *n* (%)	459 (26.9%)	123 (29.6%)	0.294
Drinking alcohol (%)	505 (27.2)	125 (29.7)	0.31
Normal defecation, *n* (%)	1,481 (86.7%)	281 (67.5%)	<0.001
Metabolic syndrome, *n* (%)	914 (53.5%)	243 (58.4%)	0.079
Surgery, *n* (%)	288 (16.9%)	98 (23.6%)	0.0019
Time, hours, mean (SD)	4.9 (5.0)	4.5 (4.6)	0.103
Bun, mmol/L, mean (SD)	5.8 (3.0)	7.5 (4.2)	<0.001
Creatinine, μmol/L, mean (SD)	69.5 (35.3)	89.1 (70.2)	
Albumin, g/L, mean (SD)	38.5 (4.5)	36.2 (5.7)	<0.01
k, mmol/L, mean (SD)	3.9 (0.4)	3.8 (0.5)	<0.01
Total, g/L, mean (SD)	66.9 (6.9)	64.8 (9.7)	<0.01
Ast, U/L, mean (SD)	20.4 (10.8)	38.7 (25.3)	<0.01
NEWS2 score, mean (SD)	2.5 (2.3)	8.3 (2.4)	<0.01
Glasgow Coma Scale score, mean (SD)	12.9 (3.2)	6.4 (2.5)	<0.01

Fecal and/or urinary incontinence refers to the inability to control bowel movements and/or urination. Time indicates the time of hospital admission. Metabolic syndrome denotes a cluster of conditions, including hyperglycemia, dyslipidemia, and hypertension. BUN stands for Blood Urea Nitrogen. Creatinine refers to serum creatinine levels. Albumin indicates serum albumin concentration. K represents serum potassium levels. AST (Aspartate Aminotransferase), also known as serum glutamic oxaloacetic transaminase (SGOT), is a liver enzyme. Total protein refers to the total amount of protein in the serum. Surgical intervention indicates whether a patient underwent any surgical procedures.

### Feature selection

To reduce multicollinearity and select the most relevant predictive variables, we used LASSO (Least Absolute Shrinkage and Selection Operator) regression for feature selection (see Supplementary Figure 1). LASSO regression introduces an L1 regularization term, which can shrink the coefficients of unimportant features to zero, thus achieving feature selection. We utilized cross-validation to determine the optimal regularization parameter, balancing the model’s complexity and predictive performance. In this study, we initially included 17 variables as the preselected factors for the model. Through LASSO regression analysis, we ultimately selected 8 independent variables for inclusion in the model. These variables include instances of incontinence (defecation), metabolic syndrome (Metabolic Syndrome), time from onset to admission (time), serum potassium levels (k), aspartate aminotransferase levels (ast), NEWS2 score (news2), Glasgow Coma Scale score (gcs), and surgical status (surgery). This approach not only effectively reduced the number of features, enhancing the model’s interpretability and generalizability but also lowered the risk of overfitting. The refined set of selected variables represents the most influential factors on the predictive outcomes, providing more concise and effective input features for subsequent machine learning models. These chosen predictive factors encompass multiple aspects of the patients’ physiological status, clinical assessments, biochemical indicators, and therapeutic interventions.

### Model selection in machine learning

This study evaluates the performance of seven machine learning models: logistic regression (serving as the baseline model), support vector machine (SVM), Random forest, XGBoost, LightGBM, Neural networks, and Bayesian models. These models include both linear and nonlinear methods, along with various ensemble learning algorithms, to thoroughly assess the effectiveness of different machine learning approaches in predicting short-term mortality risk in patients with stroke and traumatic brain injury.

### Model evaluation methods

We adopt multiple approaches to comprehensively assess model performance. First, we utilize 10-fold cross-validation to evaluate the model’s performance across different data subsets, and we calculate the 95% confidence interval using the bootstrap method (repeated 1,000 times) to ensure result robustness. Evaluation metrics include Area Under the Curve (AUC), Recall, accuracy, F1 Score, and Precision. These metrics provide a comprehensive reflection of the model’s classification performance and predictive accuracy. To enhance the model’s interpretability, we employ SHAP (Shapley Additive explanations) value analysis to assess variable importance. SHAP values can explain the contribution of each feature to individual predictions, helping to understand the model’s decision-making process and identify key predictive factors. To evaluate the model’s generalization capability and temporal stability, we also conduct time series validation. Specifically, we divide the dataset into training and testing sets in chronological order, with a ratio of 7:3. This means we use the first 70% of the data (in temporal order) as the training set and the last 30% (approximately corresponding to the last year of data) as an independent testing set. This approach simulates the model’s performance in real-world applications, where historical data is used to predict future outcomes, thus better assessing the model’s predictive ability on new data. By combining cross-validation, bootstrap methods, SHAP value analysis, and time series validation, we can comprehensively evaluate the model’s performance, stability, interpretability, and generalization capability.

### Statistical methods

**Figure 1:**
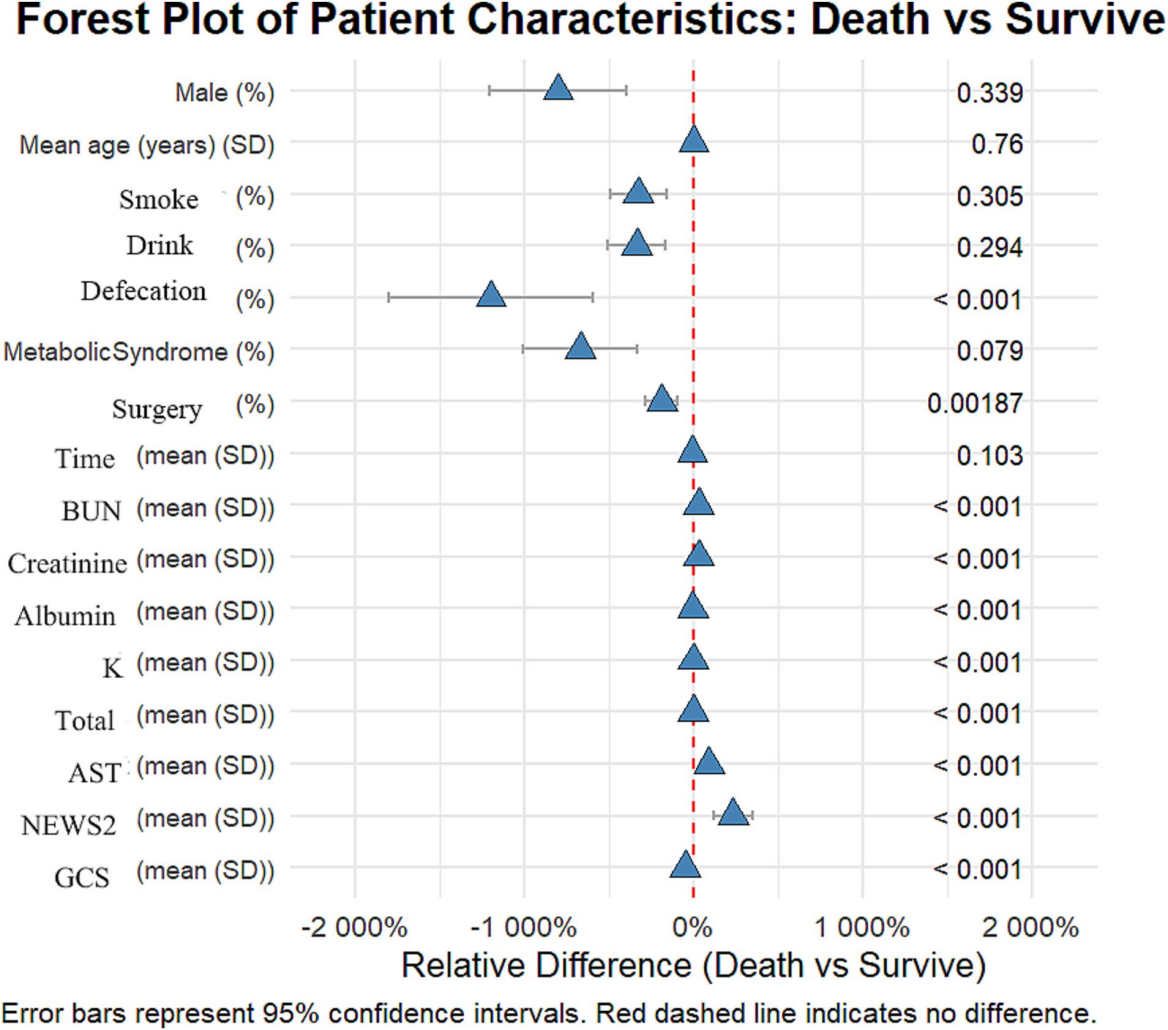
Forest plot: differences in various characteristics between the deceased group and the surviving group among patients with stroke and traumatic brain injury.

All statistical analyses were conducted using R software (version 4.3.2). Continuous variables are presented as mean ± standard deviation or median (interquartile range), depending on their distribution characteristics; categorical variables are presented as frequency (percentage). Group comparisons were performed using *t*-tests, Mann–Whitney U tests, chi-square tests, or Fisher’s exact tests, as appropriate for the data type. To visually illustrate the relationship between baseline data and in-hospital mortality, we constructed a forest plot (see Figure 1). This forest plot displays the impact of various baseline characteristics on in-hospital mortality, including odds ratios (OR) and their 95% confidence intervals, facilitating the rapid identification of potential risk factors, The forest plot displays the relative differences between survival and early mortality groups using percentage changes. The wide range of the x-axis (−2,000 to 250%) was necessary to accurately represent the substantial magnitude of differences in continuous variables between groups. This scaling allows for comprehensive visualization of both subtle and dramatic differences across all variables, though some may appear extreme due to the relative percentage calculation method. We employed LASSO regression for feature selection, with the regularization parameter determined via 10-fold cross-validation. The model performance evaluation metrics include AUC, sensitivity, specificity, accuracy, precision, and F1 score, which were obtained through 10-fold cross-validation and computed using the bootstrap method (1,000 iterations) to determine 95% confidence intervals. SHAP value analysis was conducted to evaluate variable importance. Temporal validation of the model utilized a 70:30 split of the dataset to assess its reliability. All statistical tests conducted were two-sided, with *p* < 0.05 considered statistically significant.

## Results

### Baseline characteristics

This study included 2,125 patients, with 1,709 in the survival group and 416 in the early mortality group. There were no significant differences in gender and age between the groups. The proportion of patients with traumatic brain injury (TBI) was significantly higher in the early mortality group compared to the survival group (21.9% vs. 78.1%, *p* < 0.012). Clinically, the incidence of urinary and fecal incontinence was significantly higher in the early mortality group (32.5% vs. 13.3%, *p* < 0.001), and the rate of surgical intervention was also greater (23.6% vs. 16.9%, *p* = 0.0019).

In terms of laboratory indicators, patients in the early mortality group had significantly higher levels of urea nitrogen (7.5 vs. 5.8 mmol/L) and AST (38.7 vs. 20.4 U/L) compared to the survival group, while levels of albumin (36.2 vs. 38.5 g/L), potassium (3.8 vs. 3.9 mmol/L), and total protein (64.8 vs. 66.9 g/L) were significantly lower in the early mortality group (all *p* < 0.01). Regarding scores, the NEWS2 score was significantly higher in the early mortality group (8.3 vs. 2.5), while the GCS score was significantly lower (6.4 vs. 12.9, both *p* < 0.01). These results indicate that patients in the early mortality group had a more severe overall clinical status upon admission.

Our variable selection followed a systematic multi-stage process. Initially, univariate analyses were performed to identify potentially significant variables (as shown in Figure 1). Subsequently, LASSO regression was employed for variable selection, followed by multivariate logistic regression to control for potential confounders. This stepwise approach explains why some variables (such as Metabolic Syndrome) show different significance levels across various analyses, as their statistical importance was reassessed at each stage while accounting for inter-variable relationships.

### Model development results

In predicting inpatient mortality rates for stroke and traumatic brain injury, we compared the performance of multiple machine learning models (see Table 2). The baseline model used logistic regression, which performed well, achieving an AUC of 0.973 (95% CI: 0.960–0.983), a recall of 0.963 (95% CI: 0.944–0.978), an accuracy of 0.928 (95% CI: 0.904–0.945), an F1 score of 0.955 (95% CI: 0.941–0.967), and a precision of 0.948 (95% CI: 0.926–0.965). However, the random forest model slightly outperformed logistic regression across all metrics and was ultimately selected as the optimal model. The random forest model achieved an AUC of 0.978 (95% CI: 0.966–0.986), a recall of 0.965 (95% CI: 0.946–0.980), an accuracy of 0.936 (95% CI: 0.913–0.953), an F1 score of 0.960 (95% CI: 0.946–0.972), and a precision of 0.956 (95% CI: 0.933–0.971). Notably, other advanced machine learning models such as XGBoost, LightGBM, and neural networks also demonstrated excellent performance, comparable to logistic regression and random forest. This indicates that in this study, multiple machine learning methods can effectively predict inpatient mortality, but random forest has a slight edge in overall performance. Additionally, results from time series validation (AUC 0.975, 95% CI: 0.963–0.986) indicate that the model maintains good predictive capability on new data, confirming its generalization performance.

**Table 2:** Performance comparison of machine learning models for predicting in-hospital mortality of stroke and traumatic brain injury.

Model	AUC (95% CI)	Recall(95% CI)	Accuracy (95% CI)	F1Score(95% CI)	Precision (95% CI)
Logistic	0.973(0.960–0.983)	0.963(0.944–0.978)	0.928(0.904–0.945)	0.955(0.941–0.967)	0.948(0.926–0.965)
SVM	0.956(0.930–0.964)	0.690(0.500–0.730)	0.900(0.890–0.910)	0.740(0.630–0.760)	0.800(0.730–0.840)
Random Forest	0.978(0.966–0.986)	0.965(0.946–0.980)	0.936(0.913–0.953)	0.960(0.946–0.972)	0.956(0.933–0.971)
XGBoost	0.974(0.960–0.983)	0.950(0.930–0.970)	0.928(0.904–0.945)	0.955(0.941–0.967)	0.960(0.940–0.970)
LightGBM	0.975(0.964–0.985)	0.953(0.932–0.971)	0.928(0.903–0.945)	0.955(0.940–0.967)	0.957(0.935–0.971)
Neural network	0.975(0.964–0.985)	0.953(0.932–0.971)	0.928(0.903–0.945)	0.955(0.940–0.967)	0.957(0.935–0.971)
Bayesian	0.974(0.960–0.983)	0.963(0.944–0.978)	0.928(0.904–0.945)	0.955(0.941–0.967)	0.948(0.926–0.965)
Time series validation	0.975(0.963–0.986)	0.803(0.751–0.855)	0.937(0.921–0.952)	0.825(0.799–0.850)	0.854(0.815–0.892)

CI, Confidence Interval.

### Model performance

**Figure 2:**
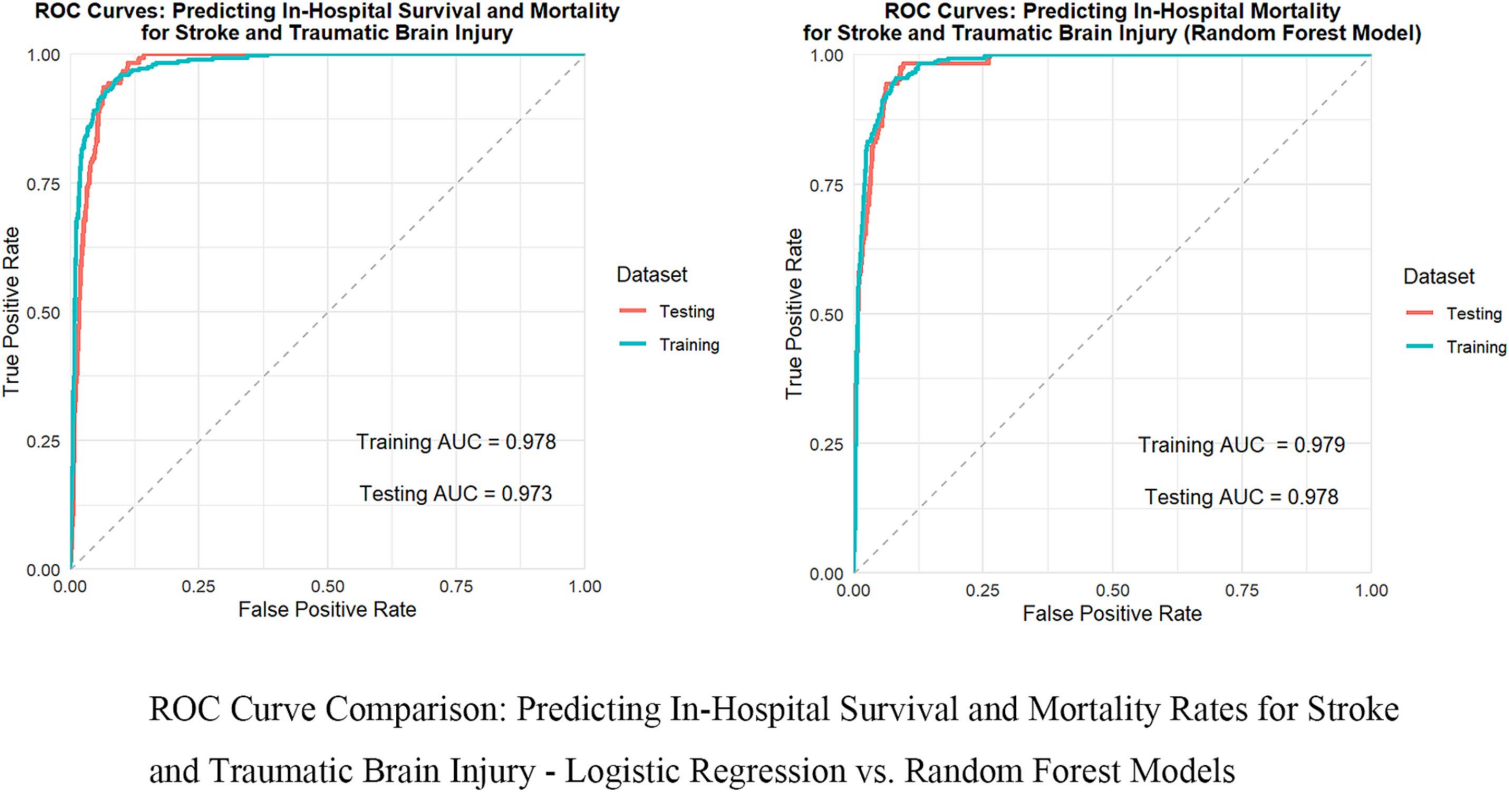
Comparison of ROC curves: in-hospital survival and mortality rates for predicting stroke and traumatic brain injury—logistic regression vs. random forest model.

**Figure 3:**
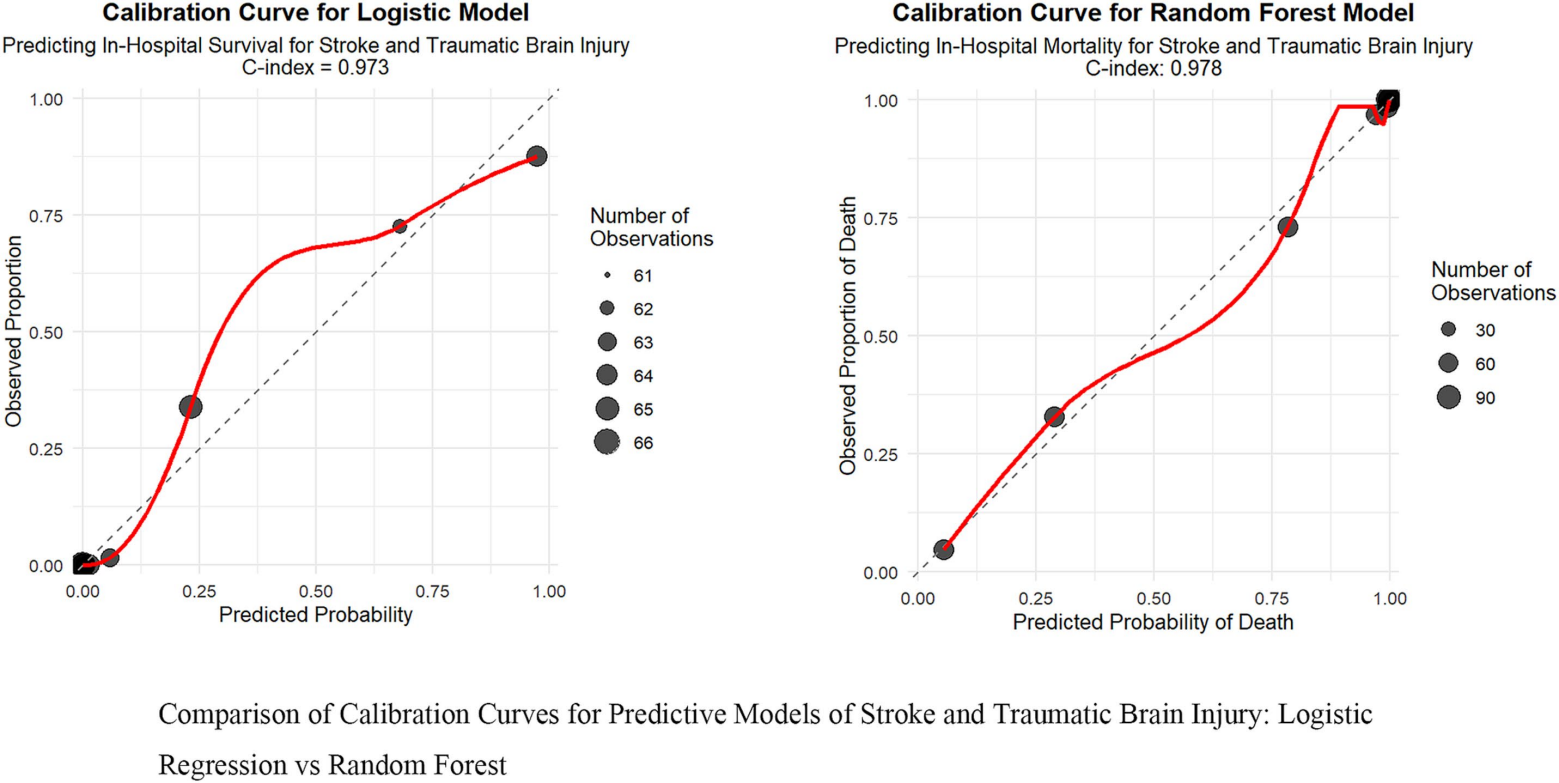
Comparison of calibration curves for predictive models of stroke and traumatic brain injury: logistic regression vs. random forest.

To thoroughly assess the model’s performance, we developed a nomogram for the Logistic regression model (see Supplementary Figure 2) and plotted the ROC curve (see Figure 2), calibration curve (see Figure 3), and DCA (Decision Curve Analysis) curve (see Supplementary Figure 3). The nomogram provides clinicians with a practical visual tool for individualized survival prediction in stroke and TBI patients. To use this nomogram, clinicians first locate the patient’s values for each predictor on their respective axes: metabolic syndrome status (present/absent), surgical intervention (yes/no), AST level, GCS score, and NEWS2 score. Each value corresponds to a point score on the topmost “Points” scale. The sum of these points is then located on the “Total points” line, and a vertical line drawn down to the bottom scale reveals the predicted probability of in-hospital survival. For example, a patient with metabolic syndrome (20 points), no surgery (0 points), AST of 40 U/L (25 points), GCS score of 9 (35 points), and NEWS2 score of 6 (44 points) would have a total score of 124 points, corresponding to a predicted survival probability of 0.073. This rapid risk assessment can assist clinicians in several ways: (1) early identification of high-risk patients requiring intensive monitoring, (2) informed decision-making regarding intervention strategies, and (3) facilitating transparent risk communication with patients and their families. The nomogram’s straightforward visual format allows for quick risk stratification within 2–3 min, making it particularly valuable in time-sensitive clinical settings. These visualization tools help in intuitively understanding the model’s predictive ability, calibration level, and clinical decision-making value. In comparing performance metrics, the random forest model slightly outperformed the Logistic regression model. The AUC for the random forest model was 0.978 (95% CI: 0.966–0.986), while the Logistic regression model was 0.973 (95% CI: 0.960–0.983). In other metrics, the random forest model also showed excellent results, with a recall of 0.965 (95% CI: 0.946–0.980), accuracy of 0.936 (95% CI: 0.913–0.953), F1 score of 0.960 (95% CI: 0.946–0.972), and precision of 0.956 (95% CI: 0.933–0.971). In contrast, while the Logistic regression model’s metrics were also high, they were slightly lower than those of the random forest model. These results indicate that, although both models demonstrated outstanding predictive capabilities, the random forest model had a slight edge in overall performance, providing more reliable support for clinical decision-making.

### Model interpretation

**Figure 4:**
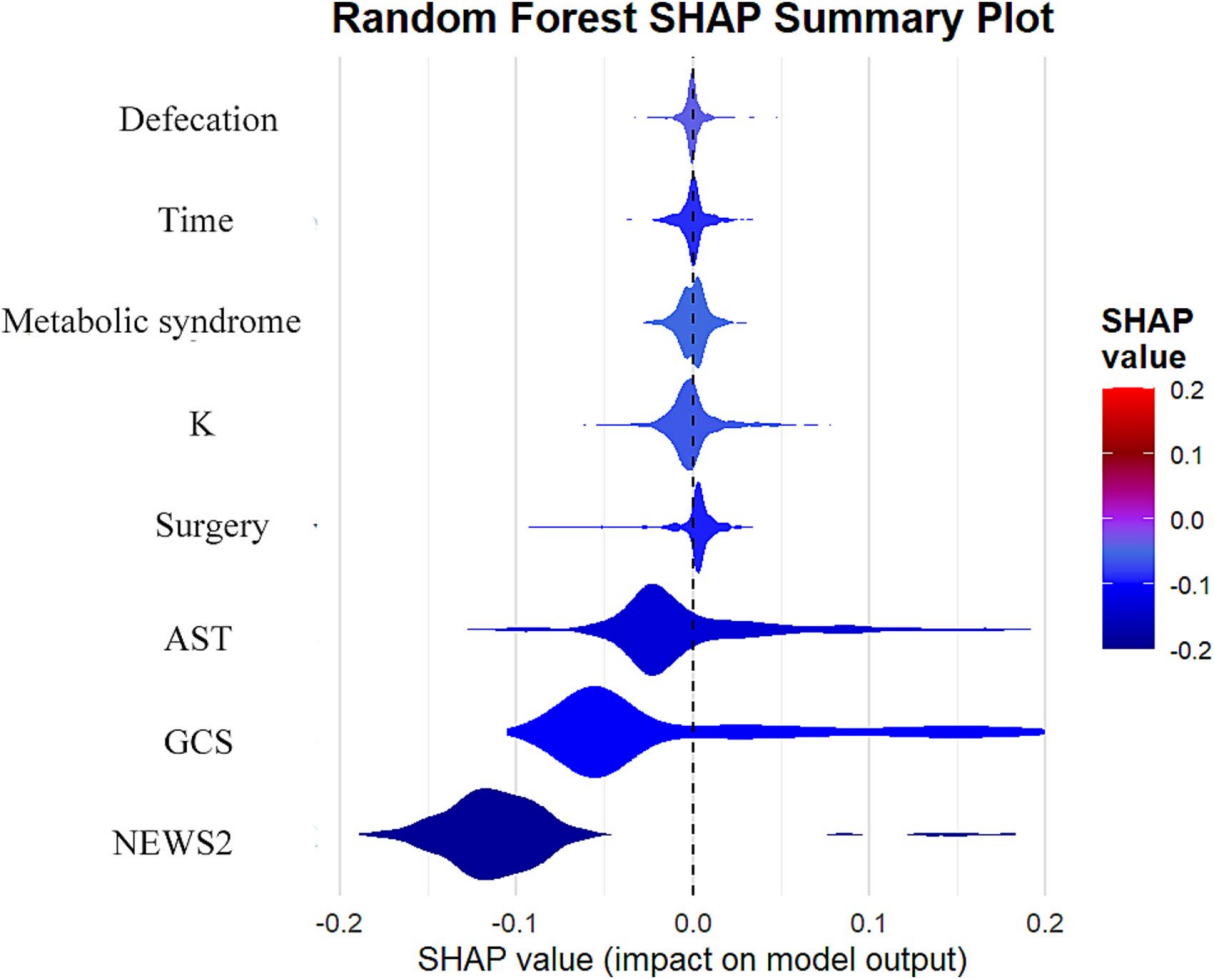
Analysis of SHAP value influences of features on prediction results in random forest models.

To better understand the decision-making process of the random forest model, we examined feature importance and SHAP values. The feature importance analysis (Supplementary Figure 4) shows that the NEWS2 score is the most crucial predictor, followed by the GCS score and AST levels. The SHAP value analysis (Figure 4) further highlights the specific impact of each feature on the predicted outcomes: higher NEWS2 scores and lower GCS scores are significantly linked to an increased risk of mortality, while elevated AST levels also show a similar trend. Although blood potassium levels, temporal factors, incontinence status, surgical status, and metabolic syndrome have relatively smaller impacts, they still provide valuable information for predictions. For instance, electrolyte disturbances, particularly potassium abnormalities, could be critical indicators in patients with cardiac dysfunction or those receiving certain medications. These findings not only enhance the model’s interpretability but also offer essential insights for clinical practice, emphasizing the importance of timely assessments of patients’ vital signs, neurological status, and liver function, which can help healthcare teams optimize patient management strategies and resource allocation. These findings not only enhance the model’s interpretability but also offer essential insights for clinical practice, emphasizing the importance of timely assessments of patients’ vital signs, neurological status, and liver function, which can help healthcare teams optimize patient management strategies and resource allocation.

## Discussion

The main findings of this study underscore the robust performance of machine learning methods, particularly the random forest model, in predicting inpatient mortality rates for patients with stroke and traumatic brain injury. Our random forest model exhibited outstanding predictive capability, achieving an AUC of 0.978 (95% CI: 0.966–0.986), significantly surpassing the traditional logistic regression model (AUC 0.973, 95% CI: 0.960–0.983). This result is not only statistically significant but also holds substantial clinical importance. The model performed remarkably well on other key metrics, including a high recall rate (0.965), accuracy (0.936), and precision (0.956), further confirming its reliability in identifying high-risk patients. The excellent performance of these metrics aligns with previous findings in research on predicting traumatic brain injury (17). Feature importance analysis reveals that the NEWS2 score, GCS score, and AST levels are key factors in predicting mortality risk, aligning well with clinical experience while offering new insights. This finding highlights the significance of clinical scores and biochemical indicators in forecasting patient outcomes. Notably, our model effectively captures the complexity of disease progression by integrating diverse predictive factors, including physiological indicators, clinical scores, and biochemical markers. This comprehensive approach not only enhances predictive accuracy but also provides extensive informational support for clinical decision-making, aligning with recent research perspectives that utilize electronic health records for deep learning (18). These findings underscore the potential of advanced machine learning techniques to improve prognostic assessments in critically ill patients, offering powerful tools for developing personalized treatment strategies and optimizing healthcare resource allocation. This conclusion further supports the significant role of machine learning in clinical decision support, resonating with recent viewpoints in the study of survival prediction models for trauma patients (19, 20).

In this study, the analysis of feature importance highlighted the critical roles of the NEWS2 score, GCS score, and AST levels in predicting the prognosis of patients with stroke and traumatic brain injury. These indicators are not only statistically significant but also carry profound clinical implications (20, 21). The NEWS2 score, as a comprehensive early warning system, sensitively reflects changes in the physiological status of patients. A higher NEWS2 score typically indicates unstable vital signs, suggesting a higher risk of mortality and poorer prognosis. The GCS score has long been regarded as the gold standard for assessing neurological function, particularly in patients with traumatic brain injury. A lower GCS score is often associated with more severe impaired consciousness and neurological deficits, directly affecting the patient’s short-term and long-term prognosis. Elevated AST levels, an important indicator of liver function, may reflect multi-organ dysfunction or liver injury, commonly seen in critically ill patients and closely linked to adverse outcomes (22).

The combination of these features offers a multidimensional assessment of the patient’s condition. The NEWS2 score reflects the overall physiological state, the GCS score focuses on neurological function, and AST levels provide insights into specific organ function. This multifaceted evaluation approach allows the model to capture the complexity of the patient’s condition more comprehensively, thereby enhancing predictive accuracy. For instance, one study indicated that combining physiological indicators with laboratory test results can significantly improve the accuracy of prognostic predictions (23, 24). Moreover, the importance of these features highlights the necessity for early intervention. Timely identification of patients with elevated NEWS2 scores can facilitate early treatment, potentially improving outcomes (25). Similarly, the dynamic monitoring of GCS scores can guide neuroprotective strategies, while monitoring AST levels aids in the prompt detection and management of potential liver dysfunction or multiple organ dysfunction.

This study utilizes an innovative methodological combination of LASSO (Least Absolute Shrinkage and Selection Operator) feature selection and Random Forest models, which offer significant advantages over traditional Logistic regression. LASSO, as a powerful feature selection tool, effectively manages high-dimensional data by automatically selecting the most relevant predictor variables while reducing the risk of overfitting through regularization (26). This feature is particularly important in our research as it helps filter out the most predictive variables from numerous potential predictors, such as NEWS2 score, GCS score, and AST levels, thereby enhancing the interpretability and generalization capacity of the model. The Random Forest model, as an ensemble learning method, offers multiple advantages compared to traditional Logistic regression. Firstly, Random Forest is capable of capturing nonlinear relationships and complex interactions between variables, which is especially crucial in complex biomedical data. Secondly, Random Forest is robust to outliers and noisy data, representing a significant advantage in clinical data analysis (27). Additionally, through the voting mechanism of multiple decision trees, Random Forest provides more stable and reliable predictive results, reducing bias that a single model might introduce (28). In our study, the Random Forest model demonstrated outstanding predictive performance, achieving an AUC of 0.978, clearly surpassing that of traditional Logistic Regression (AUC 0.973). This performance enhancement is not only statistically significant but may also lead to substantial improvements in clinical applications. For instance, more accurate risk predictions can assist physicians in better allocating medical resources and providing more timely and targeted interventions for high-risk patients.

While both our LASSO and traditional logistic regression models achieved high predictive accuracy (AUROC 0.978 and 0.973 respectively), the LASSO model was ultimately selected for its advantages in feature selection and model regularization, which enhance model parsimony and clinical interpretability. The integration of our model with existing electronic health record (EHR) systems allows for automated risk assessment using routinely collected data, including NEWS2 components, without adding manual calculation burden to healthcare providers. This automation supports standardized risk evaluation while maintaining efficient clinical workflows.

The implementation of this model in clinical practice requires addressing several practical challenges. The integration of machine learning models into clinical workflows needs consideration of both technical and operational aspects. Real-time implementation depends on appropriate infrastructure for data flow from the electronic health record (EHR) system, including automated data extraction protocols, quality control mechanisms, and strategies for handling missing data. The integration with existing EHR systems involves system compatibility, interface design, and alert mechanism implementation. To address these issues, we propose a phased implementation approach: First, developing data extraction and processing protocols for consistent data collection. Second, designing interfaces within the EHR system that provide risk predictions while maintaining workflow efficiency. Third, implementing alert systems that inform healthcare providers of high-risk patients. Fourth, establishing regular model performance monitoring and updating processes. The implementation plan includes training programs for healthcare providers and technical support systems. Future work will consider mobile application development and multi-center validation networks. This implementation approach aims to translate the model’s capabilities into practical clinical applications.

In our comparative analysis of modeling approaches, the Random Forest model showed a modest improvement in AUC (0.005) over the Logistic regression model. While this difference appears small, it reflects important methodological distinctions. The Logistic regression model, constructed with five statistically significant variables from multivariate analysis, prioritizes interpretability and clinical explicability. In contrast, the Random Forest model, utilizing eight LASSO-selected variables, demonstrates advantages in capturing non-linear relationships and complex variable interactions. Despite the minimal AUC difference, the Random Forest model exhibited superior stability and generalization across validation sets, while providing valuable variable importance rankings. We maintained both models in our analysis as they serve complementary purposes: the Logistic regression offers clear statistical interpretation and accessible risk coefficients, while the Random Forest provides more comprehensive risk assessment through its algorithmic advantages. This dual-model approach enhances clinical decision support, where even marginal performance improvements can translate to significant benefits in large-scale patient risk identification.

The Nomogram developed from the logistic regression model serves as an intuitive and convenient risk assessment tool in clinical practice. This visual representation allows clinicians to quickly estimate individual patient risk by simply locating feature values on corresponding axes. The straightforward nature of the Nomogram enhances its practical utility in clinical decision-making, making it particularly valuable for rapid risk assessment and patient communication in busy clinical settings.

Future research directions could explore more advanced machine learning approaches to enhance predictive performance. Promising algorithms include CatBoost, which excels in handling categorical features, Generalized Additive Models (GAMs) for capturing non-linear relationships, AutoML frameworks for automated model optimization, and CNN-SVR hybrid models for complex pattern recognition. Furthermore, investigating model ensemble techniques, particularly Stacked Ensembles, could potentially improve prediction accuracy by combining the strengths of multiple algorithms. Additionally, comprehensive comparisons with established clinical scoring systems, such as the Adelaide Score, would provide valuable insights into the relative performance and clinical utility of these advanced modeling approaches. These future developments could offer more robust and diverse tools for clinical decision support, ultimately enhancing risk prediction in clinical practice.

In our methodological approach, we made several key design decisions to ensure robust and comparable results. First, we deliberately used the same set of variables across all models to ensure fair comparison. While allowing each model to select its own variables might potentially enhance individual performance, our primary objective was to evaluate the models’ capabilities with identical input features. This approach effectively isolated the impact of model architecture from feature selection, providing clearer insights into each algorithm’s inherent performance characteristics, a methodology well-established in machine learning research for model comparison studies.

Regarding performance evaluation, we employed recall (sensitivity) and precision (positive predictive value) as our key metrics, as these are both mathematically equivalent to traditional medical statistics and increasingly adopted in modern medical research. This choice bridges the gap between machine learning and clinical medicine terminology while maintaining statistical rigor.

The comprehensive evaluation of seven different models across various machine learning paradigms (linear, tree-based, ensemble methods) provided valuable insights into the relationship between model complexity and performance. Notably, while some models showed similar performance metrics, this finding itself is significant as it suggests that simpler models might be sufficient for this clinical application. This comparative analysis helps clinicians and researchers make informed decisions about model selection, balancing both performance and practical implementation considerations.

### Limitations

Despite achieving favorable results in predicting the prognosis of stroke and traumatic brain injury patients, this study has certain limitations that should be considered. Firstly, although the data were sourced from two hospitals, which adds some representativeness, expanding the sample size and the number of institutions involved could yield more robust results, especially for rare prognostic events or subgroup analyses (29). Secondly, while the data from the two hospitals provide some diversity, they may still not fully reflect the medical practices and patient characteristics across a broader geographical range, which could impact the model’s generalizability (30). The study employs a retrospective design, which inherently includes limitations such as potential selection bias and information bias (31). Furthermore, although cross-validation was utilized, the lack of an independent external validation dataset remains a limitation, which is critical for assessing the model’s performance in entirely different environments (32). This research primarily focuses on short-term outcomes and does not evaluate long-term results or quality of life, both of which are equally important for patients and medical decision-making (33). Lastly, despite the model demonstrating excellent predictive performance, its clinical utility and impact on actual patient management require further validation through prospective studies (34).

Our study has several important limitations regarding potential biases and demographic representation that warrant discussion. The model’s performance may vary across different demographic subgroups, which requires careful consideration. In our analysis, we observed variations in model performance across age groups, with slightly lower prediction accuracy in elderly patients (>75 years) compared to younger adults. Gender-based analysis showed comparable performance between male and female patients, though the sample size for female patients was relatively smaller. Socioeconomic factors might also influence the model’s performance, as access to healthcare and timing of hospital admission can vary across different social groups. Additionally, our training data predominantly came from urban medical centers, which may limit the model’s generalizability to rural healthcare settings. The presence of comorbidities also affected model performance, with more complex cases showing wider confidence intervals in predictions. To address these limitations, future research should focus on: expanding the dataset to include more diverse patient populations, conducting systematic subgroup analyses across different demographic and clinical characteristics, implementing specific calibration strategies for different patient subgroups, and validating the model across different healthcare settings. Understanding these performance variations is crucial for ensuring the model’s fair and effective application across all patient populations.

This study involves the class imbalance in our patient data, with death cases accounting for approximately 20% and survival cases for 80% of the dataset. While this distribution reflects real-world clinical scenarios, it may potentially affect model performance. Although our model demonstrated satisfactory sensitivity and specificity in predicting death cases under the current data structure, we did not implement specific techniques such as SMOTE or class weights to address this imbalance. Future research could explore these data balancing techniques to potentially enhance model performance, particularly in improving prediction accuracy for minority classes and developing more robust prediction models.

A methodological limitation of this study lies in our validation approach, which utilized a single 70:30 time-based split for model validation. While this approach provided sufficient samples for model evaluation and aligned with common practices in related research, more sophisticated time series validation methods, such as rolling windows or sliding windows, could potentially offer more comprehensive assessment of model stability over time. Although our study period was relatively short with stable clinical practices and patient characteristics, future research could benefit from implementing these more dynamic validation approaches to better evaluate model performance across different time periods and ensure temporal robustness.

A limitation of our study lies in the inability to develop a Nomogram for the random forest model. Unlike logistic regression, which features linear additive relationships that can be easily visualized, random forest models incorporate multiple decision trees and complex non-linear feature interactions. These sophisticated modeling characteristics, while contributing to the model’s predictive performance, make it challenging to represent the relationship between features and outcomes through simple graphical tools like Nomograms. This highlights the inherent trade-off between model complexity and interpretability in machine learning approaches.

Our model was developed and validated using a general neurological patient population, without specific subgroup analyses for conditions such as stroke or traumatic brain injury. This approach was chosen to maintain adequate statistical power and avoid issues associated with multiple testing. While our model selection process considered both LASSO and traditional logistic regression approaches, we prioritized practical clinical utility over formal statistical testing of AUROC differences between models. Further validation studies with larger datasets will be valuable to assess the model’s performance in specific neurological conditions and confirm its generalizability across different patient subgroups.

## Conclusion

The study, utilizing emergency data, developed a robust predictive model (AUC 0.978) for assessing the prognosis of patients with stroke and traumatic brain injury by integrating LASSO feature selection and a random forest model. The research identified that readily available features in the emergency setting, such as the NEWS2 score, GCS score, and AST levels, are important predictors. This predictive tool, when integrated into electronic health record systems, can provide emergency medical teams with automated risk assessment capabilities, supporting the identification of high-risk patients and resource allocation decisions. The model leverages routinely collected clinical data to assist in clinical decision-making, such as evaluating the need for neurosurgical intervention or ICU admission. While acknowledging limitations in sample size and data sources, this study presents a practical framework for prognostic prediction in emergency care settings. Future research should focus on broadening the data scope, conducting prospective validation, and evaluating the model’s integration into clinical workflows through EHR systems to assess its impact on clinical outcomes.

In summary, this study not only advances prognostic prediction methods based on emergency data but also provides a powerful tool for enhancing the quality of emergency management for patients with stroke and traumatic brain injury, with the potential to significantly contribute to precision medicine practices in emergency care.

## Data Availability

The raw data supporting the conclusions of this article will be made available by the authors, without undue reservation.

## Supplementary material

### SUPPLEMENTARY FIGURE 1

LASSO regression model feature coefficient path diagram for stroke/TBI survival prediction, The LASSO coefficient path plot demonstrates the selection process of 17 predictive variables in the survival prediction model for stroke/traumatic brain injury patients. The x-axis represents the logarithmic values of the regularization parameter Lambda, while the y-axis shows the coefficient values of each predictive variable. Each colored curve represents the coefficient trajectory of a predictive variable as Lambda changes. The red dashed line (lambda.min) and blue dashed line (lambda) indicate the point of minimum cross-validation error and the Lambda value within one standard deviation of this minimum, respectively. Through LASSO regression variable selection, eight significant predictive variables (with non-zero coefficients) were ultimately selected at the optimal lambda value, which have substantial impact on patient survival prediction.

### SUPPLEMENTARY FIGURE 2

Nomogram for Predicting In-Hospital Survival of Stroke and TBI Patients, This nomogram is designed to predict in-hospital survival rates for stroke and traumatic brain injury (TBI) patients. It incorporates five independent predictors: Metabolic Syndrome, surgery status, aspartate aminotransferase (AST), Glasgow Coma Scale (GCS), and National Early Warning Score 2 (NEWS2). To use the nomogram, locate each patient indicator on the corresponding variable axis, draw a line upward to intersect with the Points axis to obtain a score, sum all scores to get the Total Points, and finally locate the corresponding survival probability Pr(survive) on the bottom probability axis. This nomogram provides an intuitive and convenient method for assessing patient prognosis risk.

### SUPPLEMENTARY FIGURE 3

Comparison of decision impact curves for in-hospital mortality prediction models in stroke and traumatic brain injury: logistic regression vs. random forest. The figure compares decision curves between logistic regression and random forest models in predicting in-hospital mortality for stroke and traumatic brain injury patients. The left panel shows the logistic regression model (AUC=0.973), while the right panel displays the random forest model (C-index=0.978). Each graph contains three curves: model prediction strategy (solid red line), treat-all strategy (dashed blue line), and treat-none strategy (dashed green line). The x-axis represents risk threshold probability (0-1), and the y-axis shows net benefit values. The red curves of both models significantly outperform other strategies and maintain high net benefits across most threshold ranges, indicating that both prediction models have good clinical decision-making value. The random forest model performs slightly better than the logistic regression model in high threshold ranges, providing important reference for clinicians' decision-making under different risk thresholds.

### SUPPLEMENTARY FIGURE 4

Feature Importance in Predicting In-Hospital Mortality for Stroke and Traumatic Brain Injury. The figure shows the feature importance ranking for predicting in-hospital mortality among stroke and traumatic brain injury patients. It displays importance scores for 8 key predictive features, ranked from highest to lowest: National Early Warning Score 2 (NEWS2, highest score), Glasgow Coma Scale (GCS, 95.16), Aspartate Aminotransferase (AST, 70.68), Potassium (K, 29.05), Time (17.10), Defecation Status (8.41), Surgery Status (7.34), and Metabolic Syndrome (4.54). The color gradient from deep purple to light yellow reflects the decreasing importance levels. Among these, the top three features (NEWS2, GCS, and AST) have significantly higher importance scores than the others, indicating their crucial role in predicting patient outcomes. This provides valuable reference information for clinicians in conducting risk assessments.
