# Supplementary figures and images for: A machine learning model based on emergency clinical data predicting 3-day in-hospital mortality for stroke and trauma patients
Source: Front Neurol. 2025 Mar 19;16:1512297. doi: 10.3389/fneur.2025.1512297 (PMC11966482; doi:10.3389/fneur.2025.1512297)

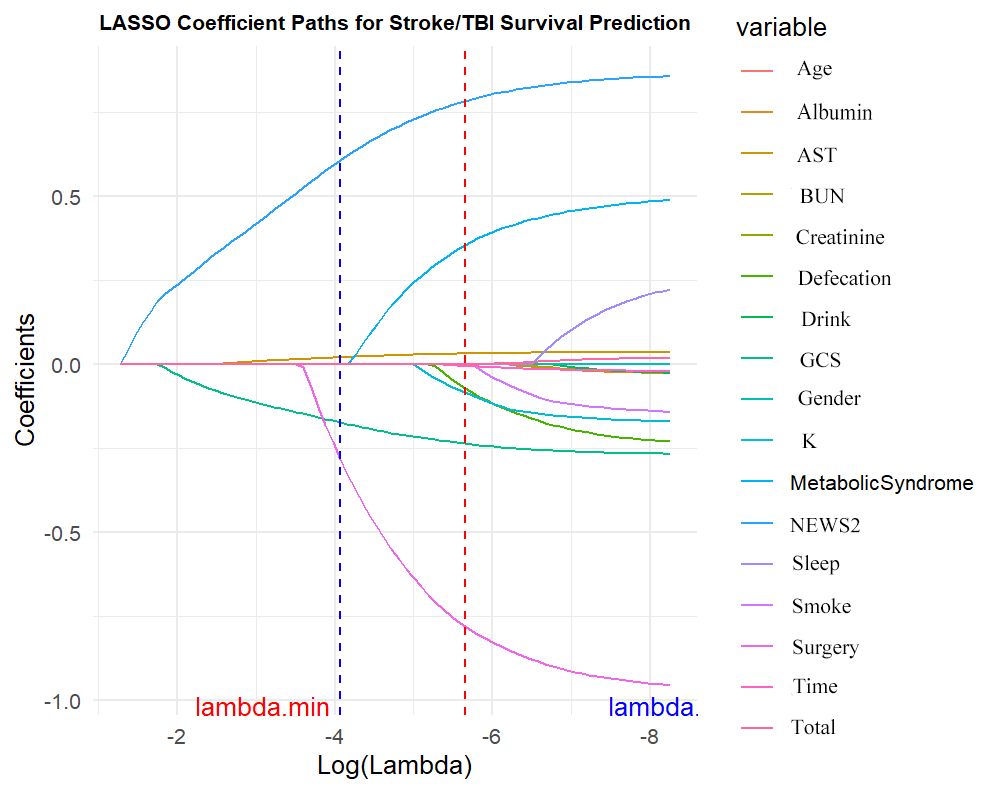

Supplement: Supplementary file 1 [file Image_1.tif]

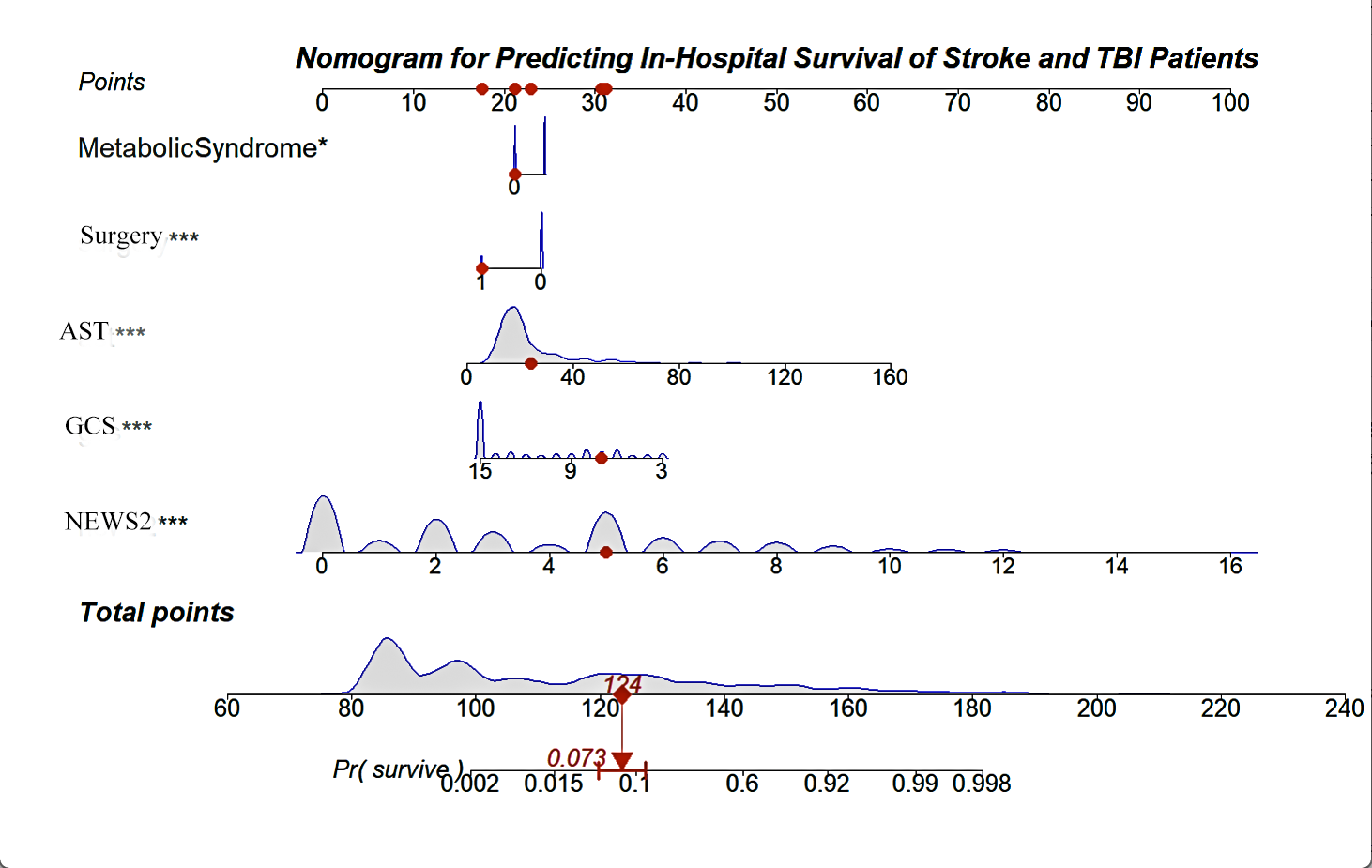

Supplement: Supplementary file 2 [file Image_2.tif]

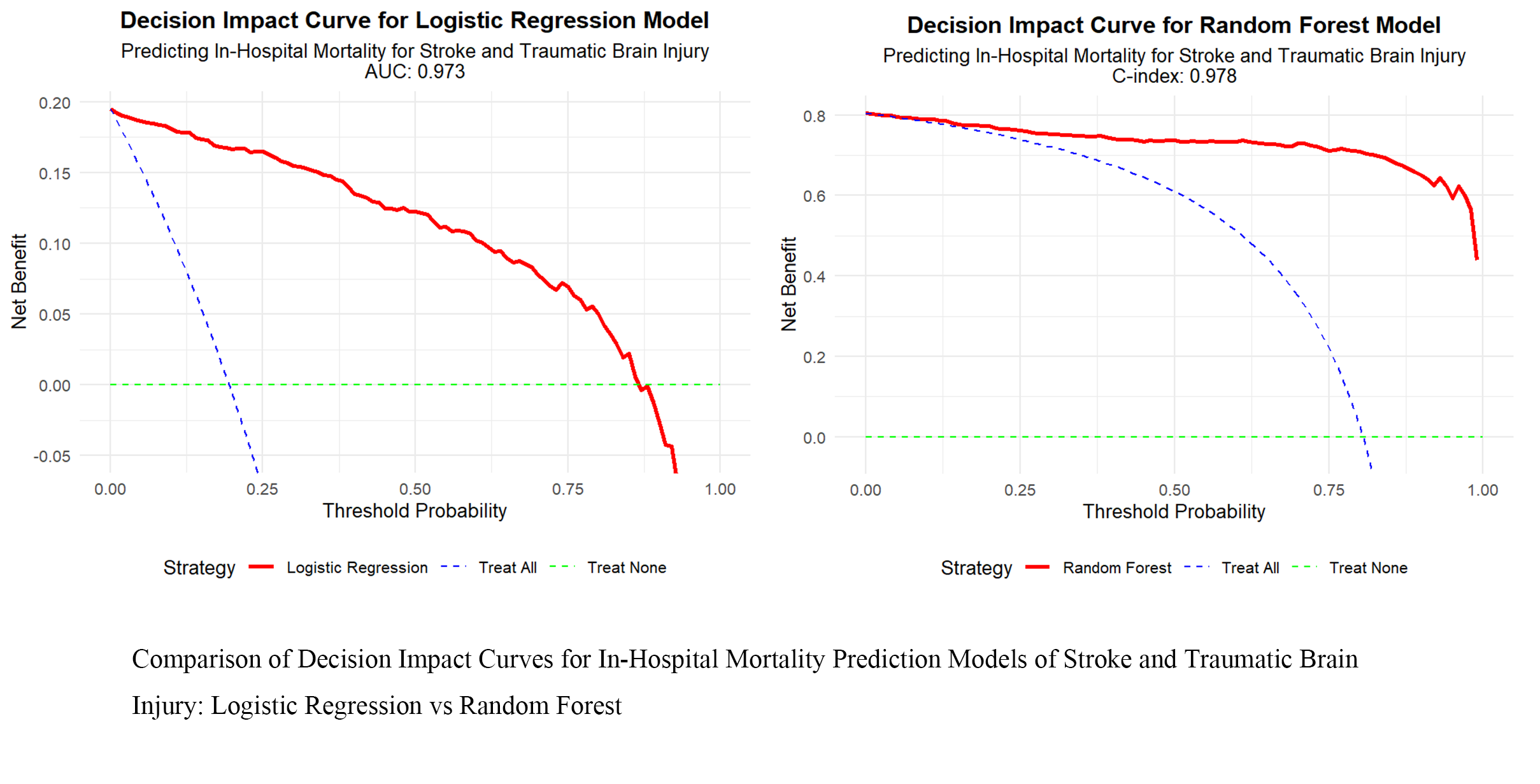

Supplement: Supplementary file 3 [file Image_3.tif]

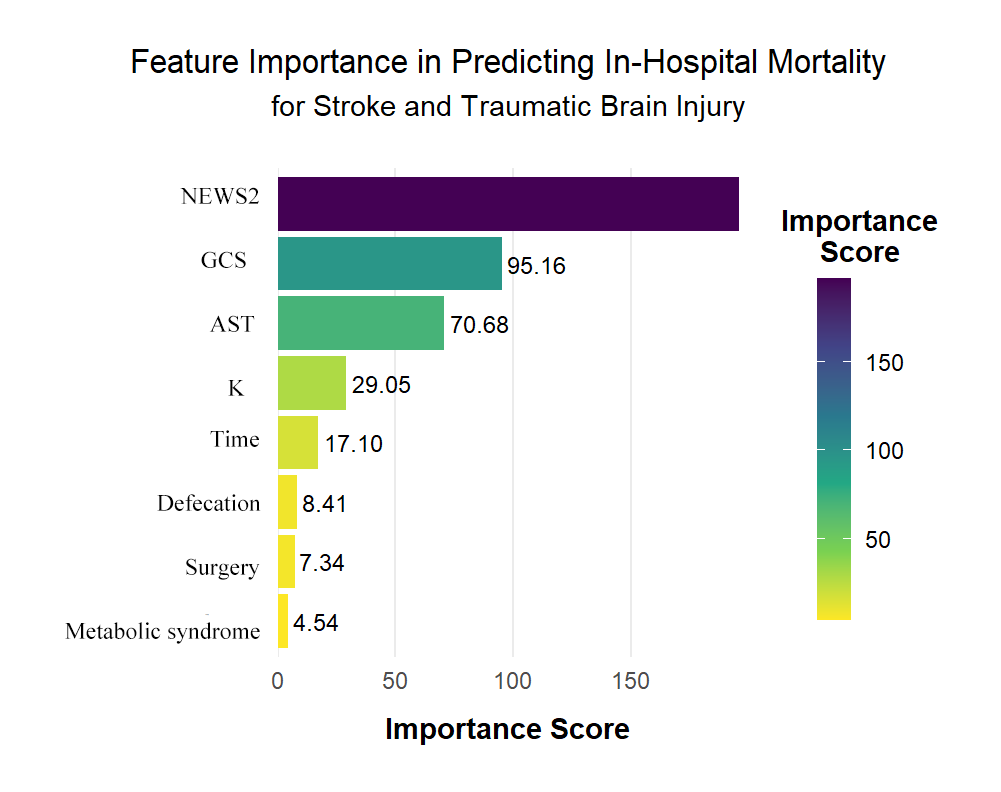

Supplement: Supplementary file 4 [file Image_4.tif]

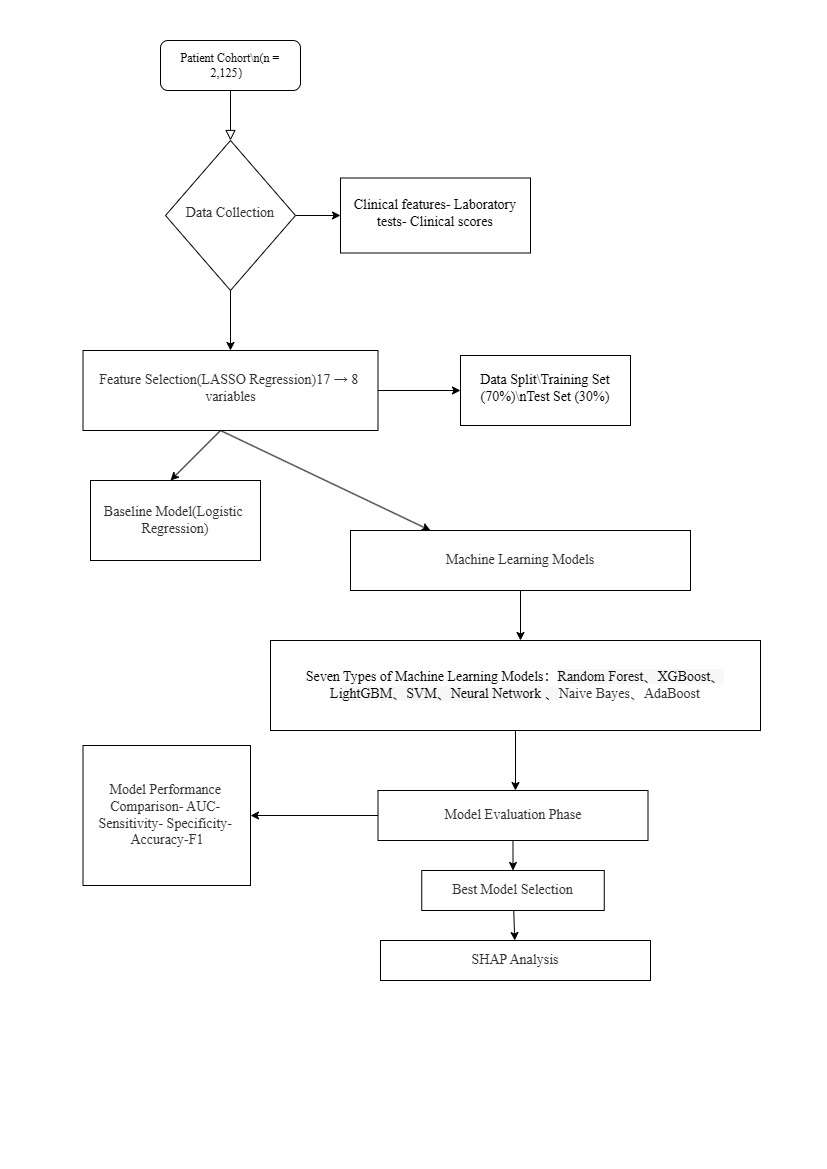

Supplement: Supplementary file 5 [file Image_5.jpeg]
