# Supplementary material for: A machine learning model based on emergency clinical data predicting 3-day in-hospital mortality for stroke and trauma patients
Source: Front Neurol. 2025 Mar 19;16:1512297. doi: 10.3389/fneur.2025.1512297 (PMC11966482; doi:10.3389/fneur.2025.1512297)
Supplement: Supplementary file 6 [file Table_1.docx]

**Hyperparameter Settings and Grid Search Ranges for Seven Machine Learning Models**

Optimal Parameter Configuration for LightGBM Model:

Learning Rate (learning_rate)

Search range: [0.01, 0.05, 0.10]

Optimal value: 0.01

Number of Leaves (num_leaves)

Search range: [31, 63]

Optimal value: 31

Maximum Tree Depth (max_depth)

Search range: [5, 10]

Optimal value: 5

Minimum Data in Leaf Node (min_data_in_leaf)

Search range: [20, 50]

Optimal value: 20

Feature Fraction (feature_fraction)

Search range: [0.8, 0.9]

Optimal value: 0.8

Grid Search Range and Optimal Values for AdaBoost Model Hyperparameters：

Number of Iterations (M)

Search Range: [50, 100, 150]

Optimal Value: 50

Maximum Depth of Decision Tree (max_depth)

Search Range: [1, 2, 3]

Optimal Value: 2

Grid Search Range and Optimal Values for SVM Model (RBF Kernel) Hyperparameters：

Kernel Function Parameter (sigma)

Search range: [0.01, 0.1, 1, 10]

Optimal value: 0.01

Penalty Parameter (C)

Search range: [0.1, 1, 10, 100]

Optimal value: 100

Grid Search Range and Optimal Values for Naive Bayes Model Hyperparameters：

Laplace Smoothing Parameter (fL)

Search Range: [0, 0.5, 1]

Optimal Value: 0

Kernel Density Estimation Switch (usekernel)

Search Range: [TRUE, FALSE]

Optimal Value: TRUE

Bandwidth Adjustment Factor (adjust)

Search Range: [0.5, 1, 1.5]

Optimal Value: 1.5

Neural Network Hyperparameter Grid Search Range and Optimal Values：

Hyperparameter Search Range

Number of Hidden Layer Neurons (size)

Search range: [5, 8, 10, 12]

Weight Decay Parameter (decay)

Search range: [0.001, 0.01, 0.1]

Optimal Parameter Values

Optimal number of hidden layer neurons (size) = 5

Optimal weight decay parameter (decay) = 0.1

Random Forest Hyperparameter Grid Search Range and Optimal Values：

Hyperparameter Search Range

mtry (number of variables selected for each split)

Search range: [2, 3, 4, 5, 6, 7, 8]

Optimal mtry = 2

min.node.size (minimum sample size for leaf nodes)

Search range: [1, 3, 5, 10]

Optimal min.node.size = 5

splitrule (split rule)

Search range: ["gini", "extratrees"]

Optimal splitrule = "gini"

XGBoost Model Hyperparameters and Search Ranges：

eta (learning_rate)

Search range: [0.01, 0.05, 0.1]

Optimal value: 0.1

max_depth

Search range: [3, 5, 7]

Best value: 7

min_child_weight

Search range: [1, 3, 5]

Optimal value: 3

gamma

Search range: [0, 0.1, 0.2]

Best value: 0.2

subsample

Search range: [0.7, 0.8, 0.9]

Best value: 0.7

colsample_bytree

Search range: [0.7, 0.8, 0.9]

Best value: 0.7
